# Supplementary material for: Evidence supported by Mendelian randomization: impact on inflammatory factors in knee osteoarthritis
Source: Front Med (Lausanne). 2024 May 28;11:1382836. doi: 10.3389/fmed.2024.1382836 (PMC11165061; doi:10.3389/fmed.2024.1382836)
Supplement: Supplementary file 1 [file Data_Sheet_1.ZIP › data/GCST90274826_5e6/826.leaveoneout.pdf]

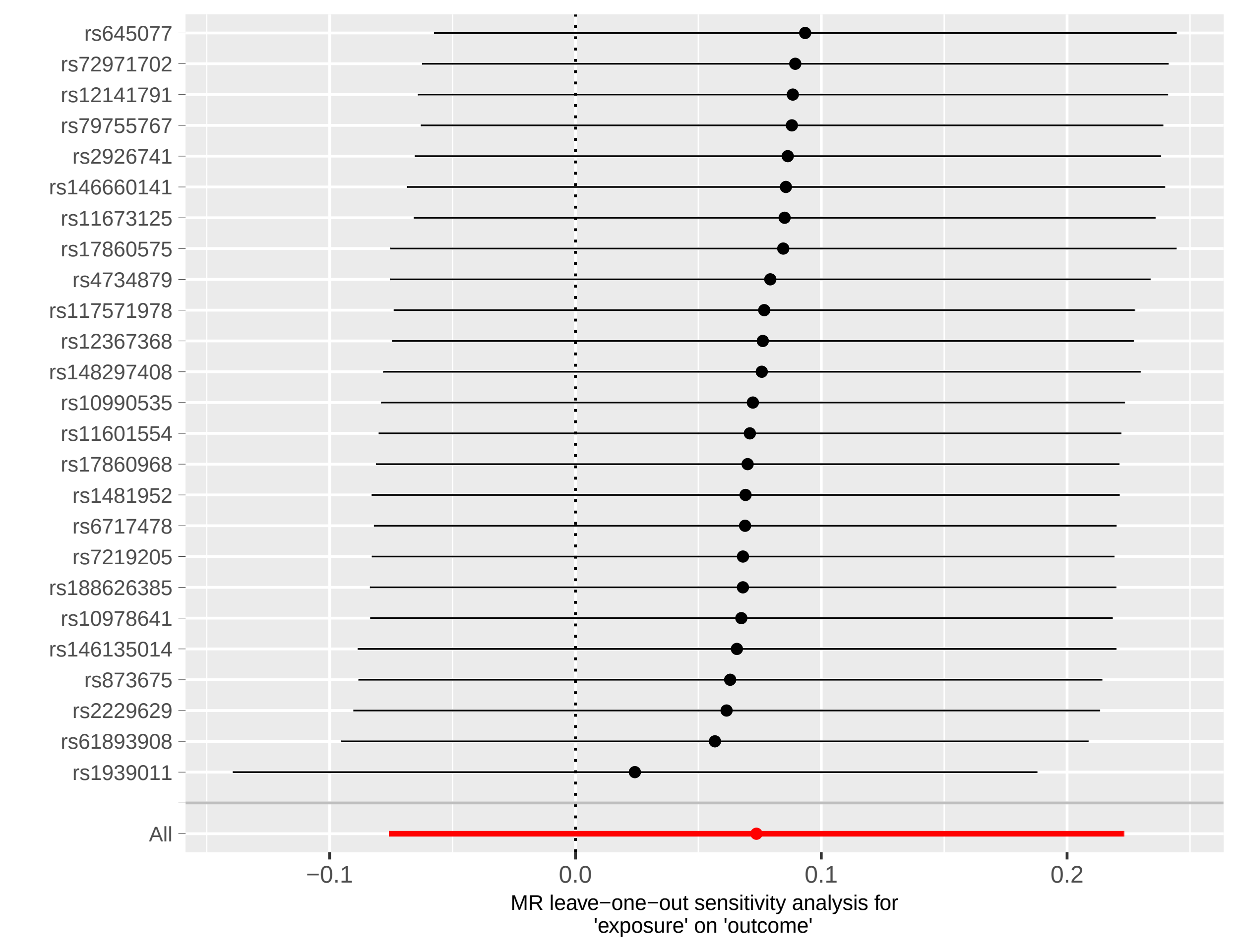
rs645077
rs72971702
rs12141791
rs79755767
rs2926741
rs146660141
rs11673125
rs17860575
rs4734879
rs117571978
rs12367368
rs148297408
rs10990535
rs11601554
rs17860968
rs1481952
rs6717478
rs7219205
rs188626385
rs10978641
rs146135014
rs873675
rs2229629
rs61893908
rs1939011
All
−0.1
0.0
0.1
0.2
MR leave−one−out sensitivity analysis for
'exposure' on 'outcome'
